# Novel Xanthomonas campestris Long-Chain-Specific 3-Oxoacyl-Acyl Carrier Protein Reductase Involved in Diffusible Signal Factor Synthesis

**DOI:** 10.1128/mBio.00596-18

**Published:** 2018-05-08

**Authors:** Zhe Hu, Huijuan Dong, Jin-Cheng Ma, Yonghong Yu, Kai-Hui Li, Qiao-Qiao Guo, Chao Zhang, Wen-Bin Zhang, Xinyun Cao, John E. Cronan, Haihong Wang

**Affiliations:** a Guangdong Provincial Key Laboratory of Protein Function and Regulation in Agricultural Organisms, College of Life Sciences, South China Agricultural University, Guangzhou, Guangdong, China; b Guangdong Food and Drug Vocational College, Guangzhou, Guangdong, China; c Department of Microbiology, University of Illinois at Urbana-Champaign, Urbana, Illinois, USA; d Department of Biochemistry, University of Illinois at Urbana-Champaign, Urbana, Illinois, USA; University of California, Berkeley

**Keywords:** *Xanthomonas*, fatty acids, quorum sensing

## Abstract

The precursors of the diffusible signal factor (DSF) family signals of Xanthomonas campestris pv. campestris are 3-hydroxyacyl-acyl carrier protein (3-hydroxyacyl-ACP) thioesters having acyl chains of 12 to 13 carbon atoms produced by the fatty acid biosynthetic pathway. We report a novel 3-oxoacyl-ACP reductase encoded by the X. campestris pv. campestris XCC0416 gene (*fabG2*), which is unable to participate in the initial steps of fatty acyl synthesis. This was shown by the failure of FabG2 expression to allow growth at the nonpermissive temperature of an Escherichia coli *fabG* temperature-sensitive strain. However, when transformed into the E. coli strain together with a plasmid bearing the Vibrio harveyi acyl-ACP synthetase gene (*aasS*), growth proceeded, but only when the medium contained octanoic acid. *In vitro* assays showed that FabG2 catalyzes the reduction of long-chain (≥C_8_) 3-oxoacyl-ACPs to 3-hydroxyacyl-ACPs but is only weakly active with shorter-chain (C_4_, C_6_) substrates. FabG1, the housekeeping 3-oxoacyl-ACP reductase encoded within the fatty acid synthesis gene cluster, could be deleted in a strain that overexpressed *fabG2* but only in octanoic acid-supplemented media. Growth of the X. campestris pv. campestris Δ*fabG1* strain overexpressing *fabG2* required *fabH* for growth with octanoic acid, indicating that octanoyl coenzyme A is elongated by X. campestris pv. campestris *fabH*. Deletion of *fabG2* reduced DSF family signal production, whereas overproduction of either FabG1 or FabG2 in the Δ*fabG2* strain restored DSF family signal levels.

## IMPORTANCE

Quorum sensing mediated by DSF signaling molecules regulates pathogenesis in several different phytopathogenic bacteria, including Xanthomonas campestris pv. campestris. DSF signaling also plays a key role in infection by the human pathogen Burkholderia cepacia. The acyl chains of the DSF molecules are diverted and remodeled from a key intermediate of the fatty acid synthesis pathway. We report a Xanthomonas campestris pv. campestris fatty acid synthesis enzyme, FabG2, of novel specificity that seems tailored to provide DSF signaling molecule precursors.

## INTRODUCTION

**FIG 1:**
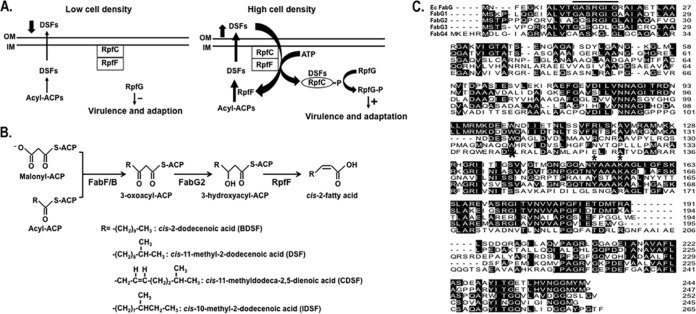
DSF family signaling cascade and 3-oxoacyl-ACP reductase candidates. (A) Schematic model of the DSF family signaling cascade. DSF quorum sensing involves a complex series of protein-protein and protein-ligand interactions that are described in the introduction. (B) Production of precursors of DSF family signals and synthesis of DSF family signals. FabF/B, long-chain 3-oxoacyl-ACP synthases; OAR, 3-oxoacyl-ACP reductase; RpfF, DSF family synthase. (C) The cofactor-binding sequence (Gly motif [GlyXXXGlyXGly]) is overlined. Catalytic triad residues (Ser-Tyr-Lys) are marked by asterisks. Alignment was constructed with Clustal W based on identical residues. OM, outer membrane; IM, inner membrane; EC, *E. coli*.

The phytopathogenic bacterium Xanthomonas campestris pv. campestris is the causal agent of black rot, which is probably the most important disease of cruciferous plants worldwide (1–3). Upon infection of the host plant, X. campestris pv. campestris produces a range of extracellular enzymes which collectively play essential roles in pathogenesis (3). The production of these factors is regulated by quorum-sensing (QS) mechanisms (Fig. 1A) mediated by the diffusible signal factor (DSF) family of fatty acids (1, 2). The first X. campestris pv. campestris DSF signal characterized was *cis*-11-methyl-2-dodecenoic acid (11-Me-C_12_:Δ^2^) (4, 5). Other DSF family signals have since been identified in X. campestris pv. campestris, including *cis*-2-dodecenoic acid (C_12_:Δ^2^; BDSF), *cis*-11-methyldodeca-2,5-dienoic acid (11-Me-C_12_:Δ^2,5^; CDSF), and *cis*-10-methyl-2-dodecenoic acid (10-Me-C_12_:Δ^2^; IDSF) (6, 7) (Fig. 1B).

In X. campestris pv. campestris, a cluster of genes designated *rpfABCDEFG* (*rpf* stands for regulation of pathogenicity factors) is involved in the biosynthesis, perception, transduction, and turnover of DSF family signaling molecules (2, 8, 9). The synthesis of DSF family signaling molecules is dependent on RpfF, an enoyl-acyl carrier protein (enoyl-ACP) hydratase/thioesterase. RpfF is a bifunctional enzyme that not only catalyzes the dehydration of 3-hydroxyacyl-ACPs to *cis*-2-enoyl-ACPs but also cleaves the acyl-ACP thioester bonds to produce free fatty acids (6, 10) (Fig. 1A). The complex pathway that regulates pathogenicity is beyond the scope of this report, and thus readers are referred to recent reviews (1, 2).

Bacteria utilize primarily a disassociated fatty acid synthase system for *de novo* production of fatty acids (11, 12). The flexible nature of this system allows the diversion of intermediates to other end products, including lipid A (13, 14), quorum-sensing signal molecules (15, 16), and vitamin cofactors (17, 18). The X. campestris pv. campestris genome contains all of the genes known to be required for fatty acid synthesis, although the synthesis mechanism has received little study. The precursors of the DSF family signals are 12- or 13-carbon 3-hydroxyacyl-ACP molecules (6, 10) derived by 3-oxoacyl-ACP reductase (OAR)-catalyzed reduction of 3-oxoacyl-ACPs (Fig. 1B). Overexpression of FabG1 led to a significant increase in the production of DSF family signals (6).

The X. campestris pv. campestris genome carries four putative OAR genes: *fabG1* (XCC1018), *fabG2* (XCC0416), *fabG3* (XCC4003), and *fabG4* (XCC0384) (Fig. 1C). XCC1018 (*fabG1*) is located within a cluster of fatty acid synthesis genes, and 69.1% of the residues of the FabG1 protein are identical to those of Escherichia coli FabG. The active-site triad (Ser, Tyr, and Lys) and the N-terminal cofactor-binding sequence defined by the E. coli FabG X-ray crystal structures are conserved in X. campestris pv. campestris FabG1 (19, 20) (Fig. 1C). Therefore, given these motifs, together with its genome location, FabG1 was considered to play the major role in the reduction of 3-oxoacyl-ACPs for the synthesis of the phospholipid fatty acyl chains. In contrast, FabG3 seems involved in the biosynthesis of xanthomonadin polyketides (21). XCC0384 (*fabG4*) is located in a putative biotin synthesis operon but contains neither the conserved catalytically active triad nor the N-terminal cofactor-binding sequence and thus seems unlikely to have OAR activity (Fig. 1C).

The remaining OAR candidate, FabG2, is encoded by a lone gene located far from the above-mentioned genes. Although alignments showed that FabG2 is only 32.4% identical to E. coli FabG, it contains the typical active-site triad and the N-terminal cofactor-binding sequence (Fig. 1C). Based on these data, it was reasonable to hypothesize that *fabG2* encodes a functional 3-oxoacyl-ACP reductase. However, given that of FabG1, the role of FabG2 seemed unlikely to be involved in bulk fatty acid synthesis. A possible FabG2 role is DSF synthesis. We report that FabG2 is a novel OAR that specifically reduces long-chain substrates.

## RESULTS

### *fabG2* encodes an OAR of novel specificity.

**FIG 2:**
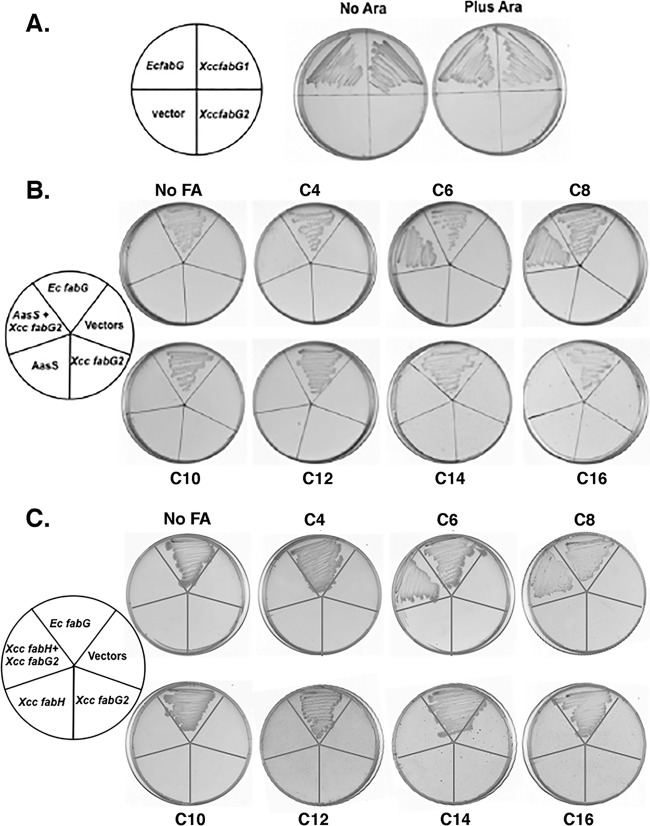
Complementation of the E. coli *fabG*(Ts) strain CL104 by expression of X. campestris pv. campestris enzymes. (A) Growth of E. coli *fabG*(Ts) strain CL104 containing plasmids that express various FabG proteins at 42°C. The FabG1, FabG2, and E. coli FabG proteins were expressed from plasmids pHZ003, pHZ004, and pTWH21, respectively, which were derived from using the compatible vectors pBAD24M and pBAD33. To allow entry of exogenous fatty acids into the E. coli fatty acid synthesis pathway, a plasmid encoding either AasS (pYFJ86) or X. campestris pv. campestris FabH (pYYH56) was used to obtain the results shown in panel B or C, respectively. “Vectors” denotes the empty vectors. No Ara, without arabinose induction; Plus Ara, arabinose induction. The medium was RB agar. (B) Growth at 42°C of E. coli strain CL104 carrying the plasmids expressing FabG2 or *E. coli fabG* in the presence or absence of AasS expression on RB plates supplemented with various fatty acids (see Materials and Methods). No FA, no fatty acid supplementation; C4, butyric acid supplementation; C6, hexanoic acid supplementation; C8, octanoic acid supplementation; C10, decanoic acid supplementation; C12, dodecanoic acid supplementation; C14, tetradecanoic acid supplementation; C16, hexadecanoic acid supplementation. The lack of growth on >C_8_ fatty acids is because their chain lengths are past the C_8_ to C_10_ branch points for synthesis of the unsaturated fatty acids required for membrane function. (C) Growth of E. coli strain CL104 containing a plasmid expressing FabG2 or E. coli FabG as in panel B, except that the expressed octanoate entry enzyme was X. campestris pv. campestris FabH in place of AasS. Fatty acid supplementation was as described for panel B.

To determine whether FabG2 has OAR activity, we transformed the E. coli *fabG*(Ts) strain CL104 with a plasmid that expressed FabG2 under arabinose control and assayed growth at 42°C. Strain CL104 lacks 3-oxoacyl-ACP reductase activity at 42°C and is unable to grow at that temperature (22). As a control, we similarly expressed the housekeeping OAR, FabG1, with the expectation that it would allow growth of strain CL104 at 42°C, and that was the case (Fig. 2A). In contrast, strain CL104 expressing FabG2 failed to grow at 42°C either in the presence or the absence of arabinose induction (Fig. 2A), and thus FabG2 seemed to lack OAR activity. However, because overexpression of FabG2 in the wild-type X. campestris pv. campestris strain Xc1 increased DSF production (see below), it seemed that FabG2 might specifically reduce 3-oxoacyl-ACPs to provide substrates for DSF synthesis and be unable to reduce short-chain 3-oxoacyl-ACPs. If so, when provided with a sufficiently long 3-oxoacyl-ACP substrate, FabG2 should functionally replace E. coli FabG. This hypothesis was tested by expressing both FabG2 and the AasS acyl-ACP synthetase (23) in E. coli CL104 and testing for growth at 42°C on plates containing octanoic acid. In this scenario, AasS converted exogenous octanoic acid to octanoyl-ACP, which was elongated to 3-oxodecanoyl-ACP. FabG2 then reduced this product to 3-hydroxydecanoyl-ACPs to allow synthesis of the fatty acids required for E. coli growth. Under these circumstances, and production of both enzymes was required (Fig. 2B).

In a second approach, we expressed both FabG2 and X. campestris pv. campestris FabH in E. coli CL104 and tested growth in the presence or absence of octanoic acid at 42°C. In this second scenario, X. campestris pv. campestris FabH condensed octanoyl coenzyme A (octanoyl-CoA) with malonyl-ACP to produce 3-oxodecanoyl-ACP (24), which FabG2 reduced to 3-hydroxydecanoyl-ACP, as described above. Fatty acid synthesis was thus primed, and growth at 42°C proceeded. Both enzymes were required (Fig. 2B). These results argued strongly that FabG2 is a 3-oxoacyl-ACP reductase that specifically reduces long-chain substrates.

To test whether a specific fatty acid chain length was required to support growth, plates supplemented with 100 µg/ml of straight-chain saturated fatty acids with chain lengths of C_4_ to C_16_ were tested (Fig. 2B and C). Only the C_6_ and C_8_ fatty acids supported the growth of derivatives of strain CL104 that expressed FabG2 plus either AasS or X. campestris pv. campestris FabH (Fig. 2B and C). The failure of butyric acid to support growth can be attributed to the inability of AasS to use this substrate (16) and/or the weak activity of FabG2 with 3-oxohexanoyl-ACP observed *in vitro* (see below). Longer fatty acids (>C_8_) failed to support the growth of >C_8_ acids because they feed into the pathway past the branch point for unsaturated fatty acid synthesis (see below).

### FabG2 preferentially reduces long-chain 3-oxoacyl-ACPs *in vitro.*

Recombinant hexahistidine-tagged FabG2 was expressed in E. coli and purified to homogeneity (see Materials and Method). Purified FabG2 had the size expected from the sequence of the tagged protein (26.4 kDa) (see Fig. S1A in the supplemental material). Size exclusion chromatography indicated that FabG2 is a multimer (trimer or tetramer) in solution (Fig. S1B).

10.1128/mBio.00596-18.1 FIG S1  Characterization and activity of the X. campestris pv. campestris FabG proteins. Download FIG S1, DOCX file, 0.1 MB. Copyright © 2018 Hu et al. 2018 Hu et al. This content is distributed under the terms of the Creative Commons Attribution 4.0 International license.

**FIG 3:**
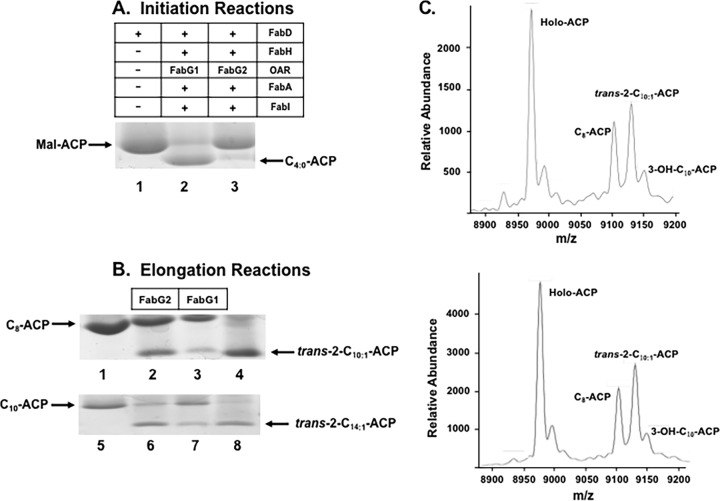
Enzymatic characterization of FabG2. (A) X. campestris pv. campestris FabG2 functions in the first cycle of fatty acid biosynthesis. Abbreviations: Mal-ACP, malonyl-ACP; C_4:0_-ACP, butyryl-ACP; FabD, E. coli ACP *S*-malonyltransferase; FabH, E. coli 3-oxoacyl-ACP synthase III; FabA, E. coli 3-hydroxyacyl-ACP dehydratase; FabI, E. coli enoyl-ACP reductase; OAR, 3-oxoacyl-ACP reductase. (B) FabG2 functions in the elongation reactions of fatty acid biosynthesis. C_8_-ACP, octanoyl-ACP; C_10_-ACP, decanoyl-ACP; *trans*-2-C_10:1_-ACP, *trans*-2-decenoyl-ACP; *trans*-2-C_12:1_-ACP, *trans*-2-dodecenoyl-ACP. Lanes 2, 3, 6, and 7 all contained FabD, FabB (E. coli 3-oxoacyl-ACP synthase I), and FabA. Lanes 1, 4, 5, and 8 contained standards. (C) MALDI-TOF MS analysis of the products of the FabG2 reaction using octanoyl-ACP as the substrate. (Top) Mass spectrum of the reaction mixture containing FabG1; (bottom) mass spectrum of the reaction mixture containing FabG2. The calculated mass values for holo-ACP, C_8_-ACP, *trans*-2-C_10:1_-ACP, and 3-OH-C_10_-ACP are 8,980, 9,106, 9,132, and 9,150, respectively.

To study FabG2, the initiation reactions of fatty acid synthesis were reconstructed from the purified E. coli proteins FabD, FabA, FabI, and FabH plus an X. campestris pv. campestris OAR (either FabG1 or FabG2). The reaction products were analyzed by conformationally sensitive gel electrophoresis (Fig. 3A). Use of the complete fatty acid synthesis cycle avoided unstable intermediates and reversible reactions. FabD converted malonyl-CoA to malonyl-ACP, and FabH reacted with acetyl-CoA to produce 3-oxobutyryl-ACP. The OAR reduced 3-oxobutyryl-ACP to 3-hydrobutyryl-ACP. Dehydration by FabA gave *trans*-2-crotonyl-ACP, which FabI reduced to butyryl-ACP, a stable product. As expected, FabG1 addition resulted in robust butyryl-ACP synthesis (Fig. 3A, lane 2), whereas FabG2 gave only trace amounts of butyryl-ACP (Fig. 3A, lane 3). Thus, FabG2 only very weakly reduces 3-oxobutyryl-ACP, consistent with its inability to support the growth of E. coli CL104 at 42°C (Fig. 2).

The successful octanoic acid supplementation argued that FabG2 was an OAR active with medium-chain-length substrates (Fig. 3B). To test this *in vitro*, we incubated E. coli FabB with malonyl-ACP and octanoyl-ACP or dodecanoyl-ACP to give 3-oxodecanoyl-ACP or 3-oxotetradecanoyl-ACP, respectively. Addition of either FabG1or FabG2 and FabA to these reaction mixtures gave a mixture of 3-hydroxyacyl-ACP and enoyl-ACP species (Fig. 3B). These products were more definitively analyzed by mass spectrometry (MS). The mass peaks (*m/z*) formed with octanoyl-ACP and either FabG2 or FabG1 were similar. These were holo-ACP (mass, 8,980) and octanoyl-ACP (mass, 9,106). The 3-hydroxdecanoyl-ACP generated a new peak at a mass of 9,150, whereas FabA dehydration of 3-hydroxdecanoyl-ACP produced a mixture of *trans*-2- and *cis*-3-decenoyl-ACP (mass, 9,132). Similar results were obtained when X. campestris pv. campestris FabH, octanoyl-CoA, and malonyl-ACP replaced octanoyl-ACP.

The substrate specificity of FabG2 was assayed by substrate-dependent reduction of NADPH absorbance at 340 nm in reaction mixtures containing holo-ACP, malonyl-CoA, NADPH, E. coli FabD and FabB, X. campestris pv. campestris FabG2, and various acyl-ACPs (C_2_ to C_14_). FabG2 displayed weak activity for 3-oxobutyryl-ACP and 3-oxohexanoyl-ACP but robust activity for long-chain 3-oxoacyl-ACPs. The most active FabG2 substrate was 3-oxodecanoyl-ACP (Table S3), and we determined the kinetics of FabG2 with this substrate. The maximal rate of NADPH reduction of 3-oxodecanoyl-ACP by FabG2 (388.8 ± 35.1 µmol min^−1^ µg^−1^) was higher than that of FabG1 (287.4 ± 34.5 µmol min^−1^ µg^−1^) with this substrate, whereas FabG2 had a lower *K*_*m*_ value (141.6 ± 20.7 µM) than X. campestris pv. campestris FabG1 (214.3 ± 32.5 µM). The *K*_cat_ values for FabG1 and FabG2 were 29.0 ± 6.0 s^−1^ and 40.9 ± 8.1 s^−1^, respectively.

### Overexpression of FabG2 plus supplementation with octanoic acid allows deletion of the *fabG1* gene.

The physiological functions of FabG2 were tested by disruption of *fabG1* and *fabG2* using suicide plasmids carrying in-frame gene deletions (Fig. S2). A *ΔfabG2* deletion strain was readily generated (Fig. S2C), but no *fabG1* deletion strain could be isolated. Only the single-crossover integrant strain HZ1 was obtained (Fig. S2), which indicated that X. campestris pv. campestris *fabG1* is essential. However, since FabG2 restored E. coli CL104 growth in the presence of exogenous octanoic acid, we plated the *fabG1* single-crossover integrant (strain HZ1) on medium containing octanoic acid to allow the second crossover to give a *fabG1* deletion, but this failed. Arguing that differential expression levels of the two genes might explain this failure, we measured their transcription and found that *fabG1* transcription was 5- to 7-fold higher than that of *fabG2* (data not shown). Given these data, we overexpressed FabG2 using the vector pSRK-Gm (25) in the single-crossover strain and selected for growth in the presence of octanoic acid, which produced the Δ*fabG1* strain HZ6 (Δ*fabG1/*p*fabG2*) (Fig. S2F).

10.1128/mBio.00596-18.2 FIG S2  Strategy for isolation of X. campestris pv. campestris *fabG* deletion strains and validation of *fabG* mutant strains. Download FIG S2, DOCX file, 0.1 MB. Copyright © 2018 Hu et al. 2018 Hu et al. This content is distributed under the terms of the Creative Commons Attribution 4.0 International license.

**FIG 4:**
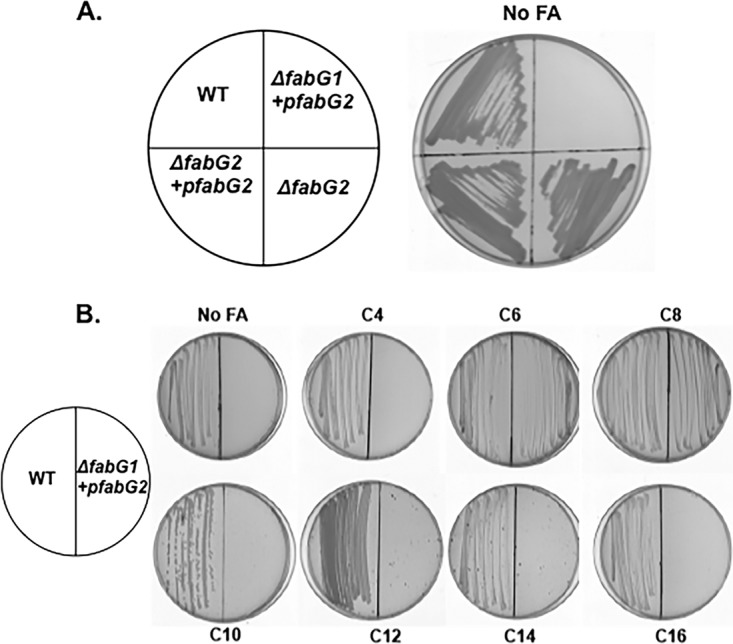
Growth of X. campestris pv. campestris Δ*fabG* strains on supplemented or unsupplemented NYG plates. (A) Growth of *ΔfabG* strains. WT, wild-type X. campestris pv. campestris strain Xc1; Δ*fabG1*+p*fabG2*, pHZ009 strain. (B) Complementation of the Δ*fabG1*strain by plasmid-borne *fabG2* in the presence of various fatty acid supplements. Designations: WT, wild-type strain Xc1; *ΔfabG1+pfabG2*, Δ*fabG1* mutant strain carrying *fabG2*plasmid pHZ009. Fatty acid designations are as described for Fig. 2.

**FIG 5:**
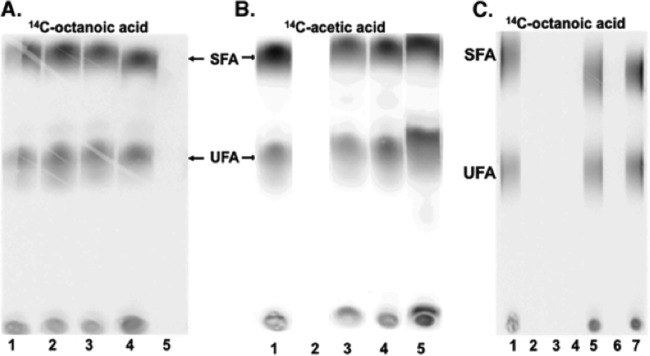
Incorporation of [1-^14^C]octanoate or [1-^14^C]acetate into methyl esters derived from the phospholipid fatty acids of X. campestris pv. campestris or E. coli strains (see Materials and Methods). (A to C) Autoradiograms of argentation thin-layer chromatographic analyses of X. campestris pv. campestris strains (A and B) and E. coli strains (C). The labeled precursor is given above the autoradiograms. (A and B) Lane 1, wild-type strain Xc1; lane 2, *ΔfabG1* strain expressing FabG2 from pHZ009; lane 3, *ΔfabG2* strain HZ3; lane 4, *ΔfabG2* strain expressing FabG2 (strain HZ4); lane 5, X. campestris pv. campestris Δ*fabH* complemented with E. coli *fabH* (strain T-3). (C) E. coli *fabG*(Ts) strain CL104 derivatives were labeled at 42°C. Lane 1, plasmid pTWH21 encoding E. coli FabG plus the vector pBAD33; lane 2, vectors pBAD24M and pBAD33; lane 3, plasmid pYFJ86 expressing V. harveyi AasS plus the vector pBAD24M; lane 4, plasmid pHZ004 expressing FabG2 plus the vector pBAD33; lane 5, plasmids pHZ004 and pYFJ86 expressing FabG2 and AasS, respectively; lane 6, plasmid pYYH56 expressing X. campestris pv. campestris FabH plus the vector pBAD24M; lane 7, plasmids expressing FabG2 and X. campestris pv. campestris *fabH* (pHZ004 and pYYH56, respectively). SFA, saturated fatty acid; UFA, unsaturated fatty acid.

Deletion of *fabG2* did not affect growth on NaCl-yeast extract-glycerol (NYG) plates, whereas the Δ*fabG1/*p*fabG2* strain that overproduced FabG grew only when the plates contained octanoic acid (Fig. 4AB; Fig. S3A). Our finding that the *ΔfabG1*/p*fabG2* strain (HZ6) grew when provided with octanoic acid or (less so) with hexanoic acid in the absence of AasS expression argued that X. campestris pv. campestris contained an enzyme that converted the C_6_ and C_8_ acids to the ACP thioesters required to enter the fatty acid synthesis pathway. In Pseudomonas aeruginosa PA3286a, novel 3-oxoacyl-ACP synthase III condenses malonyl-ACP with β-oxidation-derived acyl-CoAs of medium-chain lengths (C_6_ to C_8_) to produce longer-chain 3-oxoacyl-ACPs that prime fatty acid synthesis (26). Since *in vitro* X. campestris pv. campestris FabH uses octanoyl-CoA in place of octanoyl-ACP (24) and X. campestris pv. campestris encodes two acyl-CoA synthetases, RpfB and FadD (XCC1017), X. campestris pv. campestris may have an enzyme functionally analogous to PA3286. This was tested by use of a *ΔfabG2* derivative of a strain in which E. coli FabH replaced X. campestris pv. campestris FabH (24). E. coli FabH cannot accept octanoyl-CoA (27), and hence this strain is unable to incorporate labeled octanoate into long-chain fatty acids. In contrast, X. campestris pv. campestris strains expressing X. campestris pv. campestris FabG2 as the sole FabG elongated [1-^14^C]octanoic acid, but not [1-^14^C]acetic acid, whereas the X. campestris pv. campestris ΔfabH strain expressing E. coli FabH elongated only [1-^14^C]acetic acid. The wild-type strain Xc1 elongated both precursors (Fig. 5). Hence, X. campestris pv. campestris FabH is responsible for the entry of octanoic acid into the long-chain fatty acid synthesis pathway.

10.1128/mBio.00596-18.3 FIG S3  Growth of the *ΔfabG1* strain with FabG2 overexpression and effects of oleic acid supplementation. Download FIG S3, DOCX file, 0.1 MB. Copyright © 2018 Hu et al. 2018 Hu et al. This content is distributed under the terms of the Creative Commons Attribution 4.0 International license.

Growth of the Δ*fabG1/*p*fabG2* strain on plates supplemented with other fatty acids (Fig. 4B) was also tested. As seen when E. coli CL104 complemented with FabG2 was tested (Fig. 2), fatty acids (>C_8_) were unable to support growth. Presumably, this was due to a lack of unsaturated fatty acid synthesis. Indeed, when the Δ*fabG1/*p*fabG2* strain was plated with decanoic acid and oleic acid supplementation, the strain grew (Fig. S4B) and both fatty acids were required. Hence, the lack of unsaturated fatty acid synthesis is indeed responsible for the inability of fatty acids (>C_8_) to support the growth of the Δ*fabG1/*p*fabG2* strain.

To test whether FabG2 has long-chain 3-oxoacyl-ACP reductase activity in its native bacterium, we analyzed the fatty acid compositions of strain HZ6 (Δ*fabG1/*p*fabG2*) and wild-type strain Xc1 grown in NYG liquid medium containing octanoic acid by gas chromatography (GC)-MS (Table S3). The species of fatty acids produced by strain HZ6 (Δ*fabG1/*p*fabG2*) were essentially the same as those produced by the wild-type strain Xc1 grown in NYG liquid medium.

### Deletion of *fabG2* resulted in reduced production of DSF family signaling molecules.

**FIG 6:**
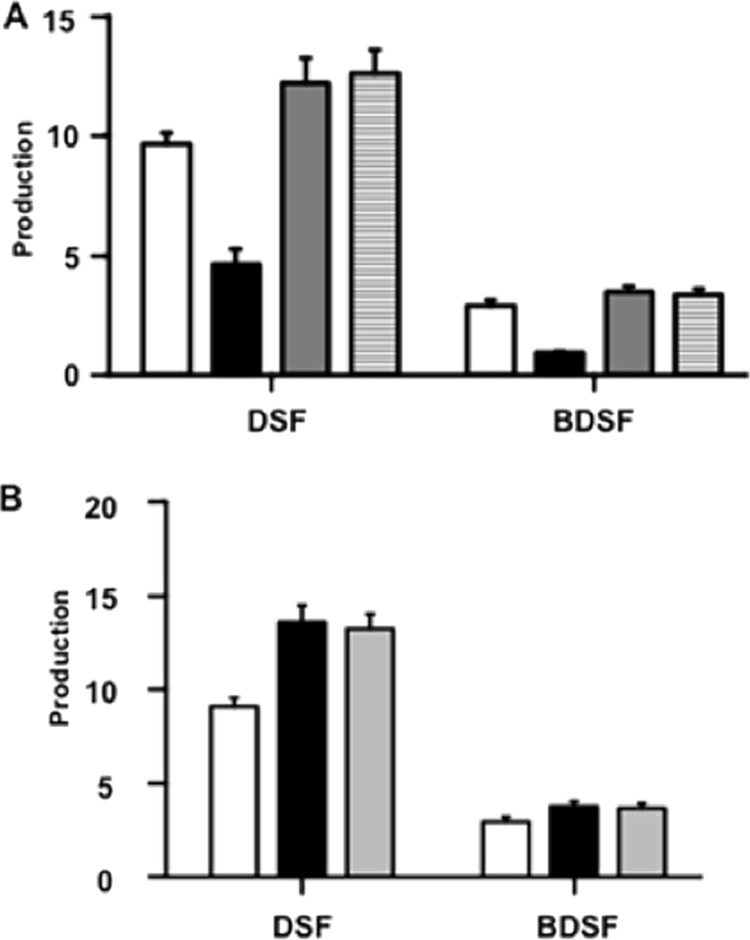
DSF signaling molecule production in Δ*fabG2* strains (see Materials and Methods). (A) Molecules produced by the Δ*fabG2* strain HZ3. White columns, wild-type X. campestris pv. campestris strain Xc1; black columns, production in the Δ*fabG2* strain; gray columns, production in the Δ*fabG2* strain carrying a plasmid encoding ΔFabG*2* (pHZ009); stippled columns, production in the Δ*fabG2* strain carrying a plasmid encoding FabG1 (pHZ013). (B) Molecules produced by strain Xc1derivatives. White columns, production in the strain carrying the vector pSRK-Gm (25); black columns, production in the strain overexpressing FabG2 (pHZ009); gray columns, production in the strain overexpressing FabG1 (pHZ013). Error bars, means ± standard deviations (*n =* 3). *, *P* < 0.05; **, *P* < 0.01; ***, *P* < 0.001, assessed with one-way analysis of variance (ANOVA). All experiments were repeated three times with similar results. Relative amounts of signal molecules were calculated based on peak areas of the detector response. Note that the amounts and relative levels of the various DSF signaling molecules are greatly affected by the medium and culture conditions (24), and hence only comparative levels are given.

3-Hydroxyacyl-ACPs of 12 or 13 carbon atoms are the precursors of the DSF family signals (Fig. 1) (6, 10). To determine whether FabG2 preferentially produces such substrates, we assayed the production of DSF family signals in the Δ*fabG2* mutant stain grown to stationary phase in NYG medium using high-performance lipid chromatography. The production of both DSF and BDSF by the Δ*fabG2* mutant stain (HZ3) was <50% of the production of wild-type strain Xc1 (Fig. 6A). Upon complementation with a plasmid expressing wild-type FabG2, the Δ*fabG2* strain increased its production of both DSF family signals (Fig. 6A), implying that FabG2 is involved in DSF family signal production. However, complementation with a plasmid overexpressing FabG1 also restored DSF family signal production to the Δ*fabG2* strain to levels similar to those produced by the strain overexpressing FabG2 (Fig. 6A). Hence, although FabG2 has a significant role in DSF family signal synthesis, it is not the sole source of 3-hydroxydodecanoyl-ACPs. Indeed, overexpression of each FabG in wild-type strain Xc1 gave DSF family signals levels 50% higher than the Xc1levels (Fig. 6B). Hence, the level of 3-oxoacyl-ACP reductase activity rather than of the specific OAR is the important parameter in the production of DSF family signals.

## DISCUSSION

In the present study, we identified FabG2 as a novel OAR that specifically reduces long-chain 3-oxoacyl-ACPs to 3-hydroxyacyl-ACPs. Unlike FabG1, FabG2 cannot replace E. coli FabG in the general fatty acid synthesis pathway. However, in the presence of an enzyme that allows exogenous fatty acids to enter the fatty acid synthetic pathway (either AasS or X. campestris pv. campestris FabH) and exogenous octanoic acid, growth was allowed at the nonpermissive temperature. Moreover, in the native bacterium, *fabG1* could be deleted only when FabG2 was overexpressed and the medium contained octanoic acid. These observations argued that the failure of FabG2 to perform all of the 3-oxoacyl-ACP reductions required for general fatty acid synthesis was due to the strain’s absent or weak ability to reduce the first 3-oxoacyl-ACP of the pathway, 3-oxobutyryl-ACP. Indeed, *in vitro* FabG2 was only weakly active with short-chain 3-oxobutyryl-ACP and 3-oxohexanoyl-ACP but readily reduced long-chain 3-oxoacyl-ACPs, with the 10-carbon substrate being the most active.

Although X. campestris pv. campestris FabG2 preferentially reduces long-chain 3-oxoacyl-ACP substrates and deletion of *fabG2* decreases the ability of X. campestris pv. campestris to produce DSF family signals, FabG2 is not required for the production of DSF family signals. Indeed, overexpression of either FabG2 or FabG1 in the wild-type strain Xc1 significantly increased the production of the DSF family signaling molecules. FabG1 is the housekeeping X. campestris pv. campestris OAR and is required for normal X. campestris pv. campestris growth, although *fabG1* can be deleted from the X. campestris pv. campestris genome provided that FabG2 is overexpressed in the presence of exogenous octanoic acid. Therefore, it seems that the role of FabG2 is to maintain a sufficiently high level of OAR activity for DSF family signal production.

## MATERIALS AND METHODS

### Materials.

Moravek supplied the radioactive precursors. Sigma-Aldrich provided *cis*-11-methyl-2-dodecenoic acid and cyclic-di-GMP. Ni-agarose columns were from Invitrogen. Agilent Technologies provided HC-C18 high-performance liquid chromatography (HPLC) columns. All other reagents were of the highest available quality. Sangon Biotechnology Co. synthesized the oligonucleotide primers.

### Bacterial strains, plasmids, and growth conditions.

The strains, plasmids, and primers used in this study are listed in Table S1 in the supplemental material. Luria-Bertani (LB) medium was used as the rich medium for E. coli growth at 37°C. Escherichia coli *fabG*(Ts) mutant strain CL104 was grown in RB medium (10 g/liter tryptone, 10 g/liter NaCl, and 1 g/liter yeast extract) (LB with one-fifth yeast extract) at 30°C (22). The X. campestris pv. campestris strains were grown in NYG medium (in grams per liter, peptone, 5; yeast extract, 3; and glycerol, 20 [pH 7.0]). Where required, antibiotics were added at the following concentrations: 100 µg/ml sodium ampicillin, 30 µg/ml kanamycin sulfate, 30 µg/ml (for E. coli) or 10 µg/ml (for X. campestris pv. campestris) gentamicin sulfate, and 50 µg/ml rifampin. l-Arabinose was used at a final concentration of 0.01%. Isopropyl-β-d-thiogalactoside (IPTG) was used at a final concentration of 1 mM. Bacterial growth in liquid medium was determined by measuring the optical density at 600 nm (OD_600_) using a Bioscreen-C automated growth curve analysis system (OY Growth Curves).

10.1128/mBio.00596-18.4 TABLE S1  Bacterial strains, plasmids, and primers. Download TABLE S1, DOCX file, 0.1 MB. Copyright © 2018 Hu et al. 2018 Hu et al. This content is distributed under the terms of the Creative Commons Attribution 4.0 International license.

### Recombinant DNA techniques and construction of plasmids.

The *fabG1* and *fabG2* PCR products were amplified from X. campestris pv. campestris strain Xc1 genomic DNA using Pfu DNA polymerase, and the primers given in Table S2 and were inserted into the T-vector plasmid pMD19 to produce plasmids pHZ001 (*fabG1*) and pHZ002 (*fabG2*). To produce plasmids pHZ003 (*fabG1*), pHZ004 (*fabG2*), pHZ005 (*fabG1*), and pHZ006 (*fabG2*), the T-vector pMD19 *fab* gene plasmids were digested with NdeI and HindIII and ligated with pBAD24M (28) or pET-28(b) digested with the same enzymes. The Δ*fabG1* and *fabG2* deletion mutant strains were constructed essentially as described previously (29). Construction details are given in the legend to Fig. S3.

10.1128/mBio.00596-18.5 TABLE S2  X. campestris pv. *campestris*FabG2 activity with various 3-oxoacyl-ACP substrates. Download TABLE S2, DOCX file, 0.1 MB. Copyright © 2018 Hu et al. 2018 Hu et al. This content is distributed under the terms of the Creative Commons Attribution 4.0 International license.

10.1128/mBio.00596-18.6 TABLE S3  Fatty acid compositions of strains Xc1, HZ3, and HZ6 grown in NYG medium. Download TABLE S3, DOCX file, 0.1 MB. Copyright © 2018 Hu et al. 2018 Hu et al. This content is distributed under the terms of the Creative Commons Attribution 4.0 International license.

### Expression and purification of plasmid-encoded proteins.

The pET28b(+)-derived plasmids carrying the various *fabG* genes were introduced into E. coli strain BL21(DE3), and the encoded proteins were expressed at high levels and purified as described previously. The enzymes were confirmed to be homogeneous using SDS-PAGE. E. coli FabD, FabH, FabZ, and FabI, Vibrio harveyi AasS, and E. coli holo-ACP proteins were purified as described previously (28). The solution structures of FabG1 and FabG2 were analyzed with size exclusion chromatography on a Superdex 200 10/300 GL column (GE Healthcare) using a model 10 AKTA purifier at 0.45 ml/min in phosphate running buffer (135 mM NaCl, 2.7 mM KCl, 1.5 mM Na_2_HPO_4_, 8 mM K_2_HPO_4_, 10% glycerol, pH 7.4), and the standards used have been described previously (30).

### Assay of FabG1 and FabG2 activities *in vitro.*

Malonyl-ACP was synthesized from holo-ACP and malonyl-CoA with E. coli FabD. Acyl-ACPs (C_6_ ACP to C_14_ ACP) were synthesized from fatty acids, ATP, and E. coli holo-ACP with AasS, as described previously (23). The reaction products were resolved with conformationally sensitive gel electrophoresis on 20% or 17.5% polyacrylamide gel containing a urea concentration optimized for the separation. The gel was stained with Coomassie brilliant blue R250.

To verify the products of the FabG2-catalyzed reaction, the acyl-ACP derivatives were purified from 500 µl of the above-described reaction mixture by the method of Zhao et al. (31). Their molecular masses were determined with matrix-assisted laser desorption ionization–time of flight (MALDI-TOF) MS (Bruker Autoflex III) as previously described (32).
