# Supplementary material for: Novel Xanthomonas campestris Long-Chain-Specific 3-Oxoacyl-Acyl Carrier Protein Reductase Involved in Diffusible Signal Factor Synthesis
Source: mBio. 2018 May 8;9(3):e00596-18. doi: 10.1128/mBio.00596-18 (PMC5941067; doi:10.1128/mBio.00596-18)
Supplement: FIG S1 [file mbo002183858sf1.docx]

**
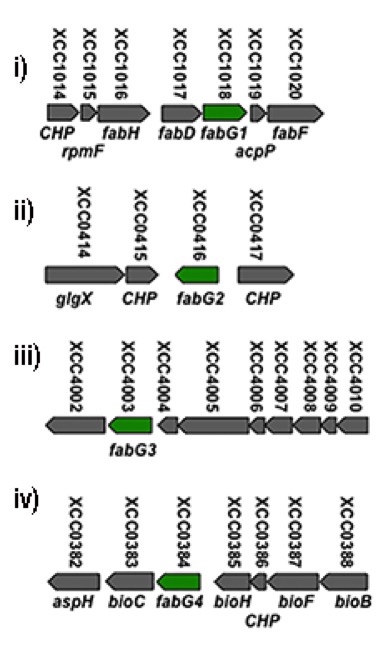
**

**Fig. S1.** Genomic contexts of the *Xcc* genes encoding putative FabG homologues. i) Gene cluster encoding the fatty acid synthesis pathway. *CHP*, gene encoding a conserved hypothetical protein; *rpmF*, gene encoding 50S ribosomal protein L32; *fabH*, gene encoding 3-oxoacyl-ACP synthase III; *fabD*, gene encoding ACP S-malonyltransferase; *acpP*, gene encoding acyl carrier protein and *fabF*, gene encoding 3-oxoacyl-ACP synthase II. ii) Location of *Xcc fabG2*. *glgX*, gene encoding isoamylase and *CHP*, gene encoding conserved hypothetical protein. iii) Xanthomonadin-biosynthesis-encoding gene cluster. iv) Biotin-biosynthesis-encoding gene cluster. *aspH*, gene encoding aspartyl beta-hydroxylase; *bioC*, gene encoding malonyl-CoA O-methyltransferase; *bioH*, gene encoding pimeloyl-(acyl-carrier protein) methyl ester esterase; *bioF*, gene encoding 8-amino-7-oxononanoate synthase and *bioB*, gene encoding biotin synthase.
