# Supplementary material for: Novel Xanthomonas campestris Long-Chain-Specific 3-Oxoacyl-Acyl Carrier Protein Reductase Involved in Diffusible Signal Factor Synthesis
Source: mBio. 2018 May 8;9(3):e00596-18. doi: 10.1128/mBio.00596-18 (PMC5941067; doi:10.1128/mBio.00596-18)
Supplement: FIG S2 [file mbo002183858sf2.docx]

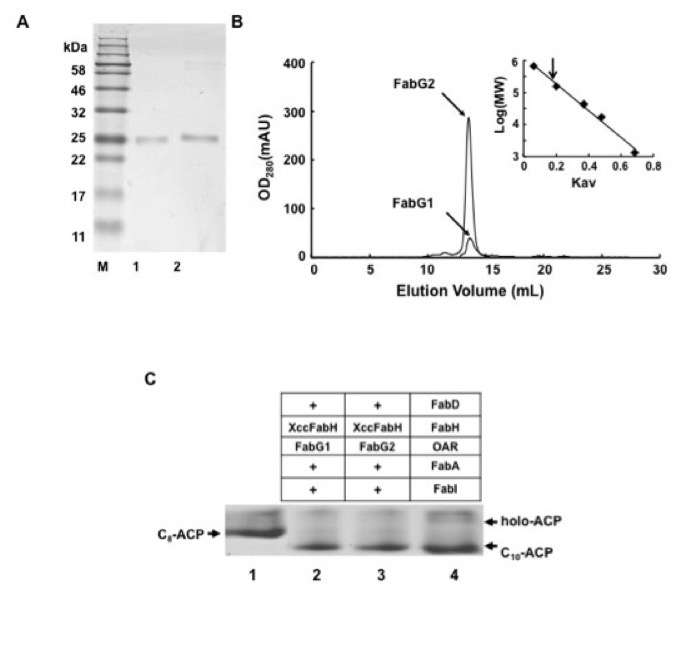


**Fig. S2. Characterization and activity of the *Xcc* FabG proteins.**

**A. Purification of *Xcc* FabG proteins by native nickel-chelate chromatography.** Lane 1, FabG1 protein. Lane 2, FabG2 protein.

**B. Size exclusion chromatography of the hexahistidine-tagged wild type FabG1 and FabG2 proteins.** The elution peaks of the molecular weight standards are given at the top of the figure.

**C. Cooperative action of FabG2 and FabH in priming fatty acid synthesis using octanoyl-CoA as substrate.** C_8:0_-ACP, octanoyl-ACP. C_10:0_-ACP, decanoyl-ACP. FabD, *E. coli* ACP S-malonyltransferase. FabH, *Xcc* 3-oxoacyl-ACP synthase III. FabA, *E. coli* 3-hydroxyacyl-ACP dehydratase. FabI, *E. coli* enoyl-ACP reductase. OAR, 3-oxoacyl-ACP reductase.
