# Supplementary material for: Novel Xanthomonas campestris Long-Chain-Specific 3-Oxoacyl-Acyl Carrier Protein Reductase Involved in Diffusible Signal Factor Synthesis
Source: mBio. 2018 May 8;9(3):e00596-18. doi: 10.1128/mBio.00596-18 (PMC5941067; doi:10.1128/mBio.00596-18)
Supplement: FIG S3 [file mbo002183858sf3.docx]

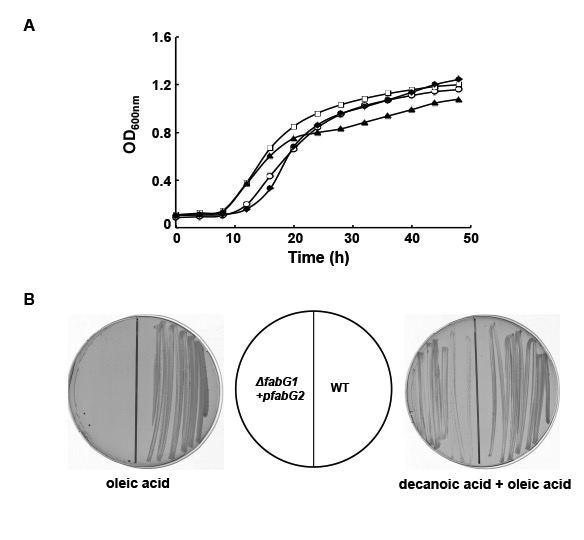


**Fig. S3. Growth of the *∆fabG1* strain with FabG2 overexpression and effects of oleic acid supplementation**

**A. Growth of the ∆*fabG1* strain and other strains supplemented with octanoic acid.** Symbols:🞏*, ∆fabG1/pfabG2* (strain HZ6); ▲*, ∆fabG2* (strain HZ3);⭘*,* wild type strain Xc1; ⧫*, ∆fabG2/pfabG2* (strain HZ4).

**B. Growth of *fabG1* mutant on NYG plates supplemented with oleic acid and decanoic acid or only oleic acid.** Designations: WT, *Xcc* wild-type strain Xc1. *ΔfabG1+pfabG2*, ∆*fabG1* carrying the *fabG2* encoding plasmid pHZ009.
