# Supplementary material for: Novel Xanthomonas campestris Long-Chain-Specific 3-Oxoacyl-Acyl Carrier Protein Reductase Involved in Diffusible Signal Factor Synthesis
Source: mBio. 2018 May 8;9(3):e00596-18. doi: 10.1128/mBio.00596-18 (PMC5941067; doi:10.1128/mBio.00596-18)
Supplement: TABLE S1 [file mbo002183858st1.docx]

**Table S1. Bacterial strains, plasmids and primers used in this study**

| Bacterial strains | Relevant characteristics*^a^* | Source |
| --- | --- | --- |
| ***E. coli*** |  |  |
| DH5α | F^-^ *deoR* *endA1 gyrA96 hsdR17*(r_K_^-^m_K_^+^) *recA1* *relA1* *supE*44 *thi-1* Δ(*lacZYA-argF*)*U*169(φ80*lacZ*ΔM15) | Laboratory collection |
| BL21 (DE3) | F^-^ *dcm ompT* *hsdS* (*r_B_*^-^ *m_B_*^-^) *gal* (λDE3) | Laboratory collection |
| S17-1 | F^-^ *thi pro* *hsdR* [RP4-2 Tc::Mu Km::Tn7 (Tp Sm)] | Laboratory collection |
| CL104 | *E.coli* *fabG*(ts), Cm^r^ Kan^r^ Tc^r^ | Laboratory collection |
| ***Xcc*** | |  |
| Xc1 | Wild-type, Rif^r^ ATCC33913 | Laboratory collection |
| HZ1 | *Xcc* Xc1 single crossover integrant of plasmid pHZ007, Rif^r^, Kan^r^ | This study |
| HZ2 | *Xcc* Xc1 single crossover integrant of plasmid pHZ008, Rif^r^, Kan^r^ | This study |
| HZ5 | HZ1 carrying plasmid pHZ009 |  |
| HZ3 | Xc1 ∆*fabG2*, Rif^r^ | This study |
| HZ4 | HZ3 carrying *Xcc* wild type *fabG2* plasmid pHZ009, Rif^r^, Gm^r^ | This study |
| HZ6 | Xc1 ∆*fabG1* carrying *Xcc* *fabG2* plasmid pHZ009, Rif^r^, Gm^r^ | This study |
| HZ25 | HZ3 carrying Xcc *fabG1* encoded plasmid pHZ013, Rif^r^, Gm^r^ | This study |
| HZ24 | HZ3 carrying vector pSRK-Gm, Rif^r^, Gm^r^ | This study |
| HZ21 | Xc1 carrying vector pSRK-Gm, Rif^r^ Gm^r^ | This study |
| HZ22 | Xc1 carrying *fabG1* plasmid pHZ013, Rif^r^ Gm^r^ | This study |
| HZ23 | Xc1 carrying *fabG2* plasmid pHZ009, Rif^r^ Gm^r^ | This study |
| **Plasmids** |  |  |
| pMD19-T | TA cloning vector, Amp^r^ | Takara |
| pBAD24M | High copy number expression vector, Amp^r^ |  |
| pBAD33 | Medium copy number vector, Cm^r^ |  |
| pET28(b) | expression vector, Kan^r^ | Novagen |
| pK18mobSacB | conjugation vector, Kan^r^ | Laboratory collection |
| pSRK-Gm | broad-host-range expression vector containing *lac* promoter and *lacI*^q^, *lacZ*α^+^, Gm^r^ | (26) |
| pSRK-Km | broad-host-range expression vector containing *lac* promoter and *lacI*^q^, *lacZ*α^+^,Kan^R^ | (26) |
| pTWH21 | pBAD24M*-EcfabG*, Amp^r^ | Laboratory collection |
| pYFJ84 | *V. harveyi* *aasS* inserted into the NdeI and BamHI sites of pET16b, Amp^r^ | (24) |
| pYFJ86 | *V. harveyi* *aasS* inserted into pBAD33, Cm^r^ | (24) |
| pYYH04 | *Xcc fabH* inserted into the NdeI and HindIII sites of pET28b, Kan^r^ | Laboratory collection |
| pYYH56 | *Xcc fabH* inserted into the NdeI and HindIII sites of pBAD43, Cm^r^ Gm^r^ | Laboratory collection |
| pHZ001 | The PCR product of *Xcc fabG1* inserted into pMD19-T, Amp^r^ | This study |
| pHZ002 | The PCR product of *Xcc fabG2* inserted into pMD19-T, Amp^r^ | This study |
| pHZ003 | *Xcc fabG1* inserted into the NdeI and HindIII sites of pBAD24M, Amp^r^ | This study |
| pHZ004 | *Xcc fabG2* inserted into the NdeI and HindIII sites of pBAD24M, Amp^r^ | This study |
| pHZ005 | *Xcc fabG1* inserted into the NdeI and HindIII sites of pET28(b), Kan^r^ | This study |
| pHZ006 | *Xcc fabG2* inserted into the NdeI and HindIII sites of pET28(b), Kan^r^ | This study |
| pHZ009 | *Xcc fabG2* inserted into the NdeI and HindIII sites of pSRK-Gm, Gm^r^ | This study |
| pHZ013 | *Xcc fabG1* inserted into the NdeI and HindIII sites of pSRK-Gm, Gm^r^ | This study |
| pHZ007 | *Xcc fabG1* in-fame deletion fragment inserted to pK18mobscaB between BamHI and HindIII sites, Kan^r^ | This study |
| pHZ008 | *Xcc fabG2* in-fame deletion fragment inserted between BamHI and HindIII sites of pK18mobscaB , Kan^r^ | This study |
| **PCR primers** | **Sequence (5'→3')** | |
| *Xcc fabG1* P2-overlap | GCATGTACATCTTGCTCATGTTGCTGTTCC | |
| *Xcc fabG1* P3-overlap | CATGAGCAAGATGTACATGCCGTGAGCGTG | |
| *Xcc fabG1* P4-HindIII | ATCAAGCTTTGACGCAGCAGCAGCCAAAT | |
| *Xcc fabG2* P1-BamHI | AATTGGATCCGATGCCCAGGTCCTTGG | |
| *Xcc fabG2* P2-overlap | CAGCCACACACCACGCCCGGCGACACTCGCATTGGT | |
| *Xcc fabG2* P3-overlap | CGAGTGTCGCCGGGCGTGGTGTGTGGCT | |
| *Xcc fabG2* P4-HindIII | AATTAAGCTTCTCGTGCAGAGCAGTTCC | |
| *pfabG2* SmaI F | TATCCCGGGCCACGTTGAGCAGATGCTCCG | |
| *pfabG2* SmaI R | TATCCCGGGAGGTGTGGACATGGCGACACT | |
| *Xcc fabG1* NdeI | TATCATATGAGCAAGCCATTGCAGG | |
