# Supplementary material for: Novel Xanthomonas campestris Long-Chain-Specific 3-Oxoacyl-Acyl Carrier Protein Reductase Involved in Diffusible Signal Factor Synthesis
Source: mBio. 2018 May 8;9(3):e00596-18. doi: 10.1128/mBio.00596-18 (PMC5941067; doi:10.1128/mBio.00596-18)
Supplement: TABLE S2 [file mbo002183858st2.docx]

**Table S2. *Xcc*FabG2 activity with various 3-oxo-acyl-ACPs**

| Substrates | XccFabG1  (μM/μg/min) | XccFabG2  (μM/μg/min) | FabG1/FabG2 |
| --- | --- | --- | --- |
| 3-oxobutyryl-ACP | 1.54±0.34 | 0.10±0.01 | 14.6 |
| 3-oxohexanoyl-ACP | 1.20±0.02 | 0.09±0.02 | 13.2 |
| 3-oxooctanoyl-ACP | 4.20±0.11 | 3.61±0.06 | 1.2 |
| 3-oxodecanoyl-ACP | 11.48±1.46 | 19.70±1.39 | 0.6 |
| 3-oxododecanoyl-ACP | 4.82±0.45 | 8.51±3.56 | 0.6 |
| 3-oxotetradecanoyl-ACP | 7.26±0.59 | 4.56±1.69 | 1.6 |
| 3-oxohexdecanoyl-ACP | 4.40±1.01 | 4.21±1.44 | 1.0 |

^α^ The values are the means ± standard deviations of three independent experiments. The statistical analyses were performed in Microsoft Excel with *P* values between each pairwise comparison calculated by two-tailed Student *t* tests. Significant differences are indicated by different letters (*P*＜0.05).
