# Supplementary material for: Novel Xanthomonas campestris Long-Chain-Specific 3-Oxoacyl-Acyl Carrier Protein Reductase Involved in Diffusible Signal Factor Synthesis
Source: mBio. 2018 May 8;9(3):e00596-18. doi: 10.1128/mBio.00596-18 (PMC5941067; doi:10.1128/mBio.00596-18)
Supplement: TABLE S3 [file mbo002183858st3.docx]

**Table S3. Fatty acid compositions of strains Xc1, HZ3 and HZ6 grown in NYG medium**

| Fatty acids | Composition (%) | | | |
| --- | --- | --- | --- | --- |
|  | **Xc1** | **HZ6** | **HZ3** | **HZ4** |
| *iso*-C14:0 | 0.82 ± 0.12 | 0.34 ± 0.01 | 0.70 ± 0.15 | 0.91 ± 0.29 |
| n-C14:0 | 1.02 ± 0.20 | 0.41 ± 0.09 | 1.05 ± 0.07 | 1.87 ± 0.47 |
| *iso*-C15:0 | 31.89 ± 0.24 | 26.16 ± 1.25 | 24.55 ± 2.68 | 37.63 ± 3.61 |
| *anteiso*-C15:0 | 19.92 ± 0.43 | 18.55 ± 1.76 | 19.48 ± 0.58 | 26.91 ± 3.70 |
| n-C15:0 | 2.11 ± 0.45 | 1.01 ± 0.01 | 1.20 ± 0.19 | 1.13 ± 0.07 |
| *iso*-C16:0 | 0.35 ± 0.10 | 0 | 6.76 ± 0.63 | 3.05 ± 0.26 |
| n-C16:1 | 14.38 ± 0.30 | 15.78 ± 1.23 | 14.39 ± 0.39 | 11.70 ± 1.35 |
| n-C16:0 | 10.60 ± 0.40 | 4.81 ± 0.32 | 7.81 ± 0.10 | 3.07 ± 0.53 |
| n-C17:0 cyclo | 8.77 ± 0.60 | 13.27 ± 0.01 | 10.80 ± 0.04 | 8.23 ± 0.60 |
| *iso*-C17:0 | 0.95 ± 0.15 | 2.32 ± 0.06 | 1.53 ± 0.12 | 0.54 ± 0.26 |
| *anteiso*-C17:0 | 6.19 ± 0.32 | 12.67 ± 0.76 | 8.31 ± 0.80 | 2.57 ± 0.83 |
| n-C17:1 | 1.73 ± 0.20 | 2.50 ± 0.16 | 1.43 ± 0.09 | 0.54 ± 0.03 |
| n-C18:1 | 0.82 ± 0.06 | 2.19 ± 0.06 | 1.21 ±0.13 | 0.27 ± 0.24 |
| n-C18:0 | 0.45 ± 0.24 | 0 | 0.78 ± 0.16 | 1.57 ± 0.87 |
|  |  |  |  |  |
| Total UFAs | 25.7 ± 0.69 | 33.74 ± 0.98 | 27.83 ± 0.93 | 20.74 ± 0.67 |
| Total BCFAs | 60.12 ± 0.84 | 60.04 ± 0.23 | 61.33 ± 0.85 | 71.61 ± 0.78 |
| Iso-BCFAs | 34.01 ± 0.40 | 28.82 ± 0.66 | 33.54 ± 0.34 | 42.13 ± 0.57 |
| Anteiso-BCFAs | 26.11 ± 0.64 | 31.22 ± 0.78 | 27.79 ± 0.54 | 29.48 ± 0.81 |

Designations: n-C14:0, myristic acid; *iso*-C15:0, 13-methyl-tetradecanoic acid; *anteiso*-C_15:0_, 12-methyl-tetradecanoic acid; n-C15:0, pentadecanoic acid; *iso*-C16:0, 14-methyl-pentadecanoic acid; n-C16:1, palmitoleic acid; n-C16:0, palmitic acid; n-C17:0 cyclo, *cis*-9,10-methylene hexadecanoic acid; *iso*-C17:0, 15-methyl-palmitic acid; *anteiso*-C17:0, 14-methyl-palmitic acid; n-C18:1, *cis*-11-octadecenoic acid; n-C18:0, stearic acid; BCFA, branched chain fatty acids; UFA, unsaturated fatty acisd.
